# Young adults’ attitudes to sharing whole-genome sequencing information: a university-based survey

**DOI:** 10.1186/s12920-019-0499-2

**Published:** 2019-04-16

**Authors:** Pepita Barnard, Sarah Sharples, Brian J. Thomson, Jonathan M. Garibaldi

**Affiliations:** 1 Department of Computer Science, Jubilee Campus, University of Nottingham, Wollaton Road, NG8 1BB Nottingham, England; 2 Horizon Digital Research Institute, Human Factors Research Group, Faculty of Engineering, University of Nottingham, University Park, NG7 2RD Nottingham, England; 3 National Institute for Health Research, Nottingham Biomedical Research Centre, Nottingham University Hospitals NHS Trust and University of Nottingham, Nottingham, England

**Keywords:** Young adults, Attitudes, Sharing, Whole-genome sequencing, Theory of planned behaviour

## Abstract

**Background:**

Genomic services are increasingly accessible to young adults starting their independent lives with responsibility for their self-care, yet their attitudes to sharing genomic information remain under-researched. This study explored attitudes of university-based 18–25 year-olds towards sharing personal whole-genome sequencing (WGS) information with relatives.

**Methods:**

We surveyed 112 young adults. Hypotheses were tested regarding the relationships between their preferences for sharing personal WGS information with relatives and factors including their gender, previous genetics-specific education, general educational attainment level and current study in a science, technology, engineering, maths or medicine (STEMM) field.

**Results:**

Most participants were positive about both their intention to share their WGS results with their parents and siblings, and their desire to know their relatives’ results. Being female and having a university-level genetics education were consistently positively correlated with intention to share one’s results with parents and with siblings as well as the desire to know relatives’ results. Additionally, females who had undertaken a genetics course at university had significantly greater intentions and desires than females who had not. Lower general educational attainment was related to a lower intention to share with siblings. Participants who were in a STEMM field had a greater desire to know their relatives’ results.

**Conclusions:**

Participants’ gender and prior genetics education were consistently related to their intentions to share WGS results with relatives and their desire to know relatives’ results. Educational attainment was found to be positively correlated with intention to share with siblings. Being in a STEMM field was related to participants’ desire to know their relatives’ results. These findings indicate that gender and genetics education are particularly important influencers on young adults’ stated sharing preferences. More research is required to examine the dependent variables studied to further understand their influence on attitudes to sharing WGS results. These findings are particularly interesting for information provision and support before genomic sequencing and post-results to improve the outcomes for individuals and their relatives.

**Electronic supplementary material:**

The online version of this article (10.1186/s12920-019-0499-2) contains supplementary material, which is available to authorized users.

## Background

### Young adults’ knowledge and attitudes to Whole-genome Sequencing

We are in the midst of a shift to genomic medicine provision. In December 2018, the UK's 100,000 Genomes Project achieved its sequencing target [1]. Personal genomic services are also increasingly accessible online, entailing less regulation and more personal choice about receiving, managing and sharing results than that offered by the UK’s National Health Service (NHS). Whole-genome Sequencing (WGS) results allow targeted and incidental information about heritability patterns. In order to support individuals in making use of this complex information, it is important to understand both their genomic understanding and their intentions. Studies regarding gender differences in the public’s knowledge of genetics present contradictory findings [2, 3]. A 2015 literature review found that students often have little genetic knowledge, frequently relying on their beliefs and affective responses to formulate their attitudes [4]. With so little genomics literature available that relates to knowledge and attitudes of young adults, further research is necessary on their knowledge and views. They are more likely to use genomic services [5], yet are limited by their genomic literacy [6]. To improve our understanding of their concerns, there clearly remains a pressing need for more research into young adults’ views of genomic testing, including exploration of factors that might, in combination, affect their use of genomic services [7]. It has also been recommended that further research into gender-related attitudinal differences among young people is needed [8, 9], to help benefit policy-making decisions and designs for genomic related services.

### Sharing whole-genome sequencing

Attitudes to WGS impact how families will use this information together, which in turn will be critical to the translation of results to health outcomes. Studies have found that the public and patients were willing to share their genetic results information with family members [10–14], with females being more likely to do so [15, 16]. Yet young adults’ attitudes towards receiving, managing and sharing genomic information have scarcely been explored. There are many issues for users of online genetic services, including which results to share with family members [7] and how to do so. In a study by Heaton and Chico [17], students and staff at a UK University appeared generally happy to forgo their own confidentiality to benefit family members with only a few reluctant to do this; yet those same few wanted to be told if their relatives had genetic test results that were pertinent to them.

### The Theory of Planned Behaviour (TPB)

The Theory of Planned Behaviour (TPB) [18, 19] has been validated and used as a theoretical framework to address family communications and information sharing with health care professionals and friends in genetic health research and initiatives [15, 16, 20]. Few studies addressing sharing genetic information refer to a theoretical framework, such as the TPB [7, 15]. Following Mackert [7], this study used the TPB to provide a framework for exploring attitudes and subjective norms. In the TPB, attitudes are defined as evaluations of individuals’ beliefs about the outcomes of given behaviours [15], while subjective norms are defined as a function of one’s beliefs about the expectations of important others and groups, weighed by one’s motivation to comply with them. Relatives are recognised as influencers of subjective norms [15].

### Research question and hypotheses

This study was designed to explore young adults’ awareness and views regarding WGS. Specifically, it aimed to answer the research question: Are there relationships between individual characteristics (such as gender, genetics course undertaken, completed educational level, field of study) and young adults’ attitudes to sharing WGS results with relatives? Hypotheses were tested to identify relationships between participants’ characteristics and their attitudes to sharing WGS results with parents and siblings, and their desire to know their relatives’ WGS results.

## Methods

### Participants

The study was approved by the School of Computer Science’s Research and Ethics Committee at the University of Nottingham. A snowball method was used to recruit participants who were students or non-academic university staff, mainly by in-person canvassing activities of the lead investigator, as well as emails to contacts, University webpage adverts and posters in University buildings. Four-hundred and fifty surveys were distributed between June and October, 2016. A voucher draw with prizes worth £40, £25 and £15 was offered to incentivise subjects to participate in the survey and a follow-up interview. All participants completed a consent form.

### Materials

The survey was available to participants on paper or in electronic form. The survey itself is provided as an additional file [see Additional file 1]. Study packs included information sheets with links to additional information about WGS, consent forms, the survey itself and envelopes for returning completed documents. The survey began with multiple choice questions about participants’ awareness of WGS, followed by a quiz on human genetics adapted from one online [21]. The main part of the survey combined questions adapted from Mackert [7] together with some original questions (shaped by the TPB [22]) to explore WGS awareness and test hypotheses about attitudes towards sharing WGS results with relatives. Although semantically differential scales, such as Likert, are often used for ease, there is no specified scale designed as a dedicated measure of factors associated with the TPB [23]. In this study, survey responses were collected using a method whereby users draw ellipses to indicate their response together with their perceived confidence in their answer [24]. This approach has not been used before in this context. In this present study, the central point of the ellipse, i.e. the mean of the endpoints, was taken as the response; the uncertainty was not used. Participants reported their gender, highest attained educational level, previous genetics education and field of study. Respondents were provided with a blank space on the questionnaire so they could self-identify their gender.

Participants reported whether they had previously undertaken a genetics course at school or at university. If both, the higher level was used for analysis. Independent variables of gender, STEMM field of study, completed educational level, and genetics course were compared to the following dependent variables: (i) intention to share WGS results with parents, (ii) intention to share with siblings and (iii) one’s desire to know relatives’ WGS results. The survey was piloted and initially validated by three international PhD students at the University of Nottingham, who completed it, commented on any difficulties or concerns regarding the questions and made recommendations that were incorporated into the final version. All survey participants were invited to comment on the survey, either inline or in the comments section at the end. Following completion of the main survey, participants were asked questions on a separate survey sheet to ascertain their views about the use of ellipses. Responses to these questions were used to assess the face validity of the use of ellipses.

All participants’ responses were manually inputted into spreadsheets. During this process a sense-checking exercise was undertaken to assess internal consistency of individuals’ responses, i.e. agreement between and among responses to survey items that reflected similar constructs, evidencing internal consistency reliability as well as criterion validity.

### Data analysis

A statistical power analysis was performed for sample size estimation. To detect a small to moderate effect (Cohen’s d = 0.4) with an estimated means SD of 2.5 on a scale from 0 to 10 and an alpha of 0.5, a sample size of 100 participants will produce power = 0.52. The dataset is provided [Additional file 2]. Two-tailed Wilcoxon-Mann-Whitney tests (U) were used for independent variables with two levels and Kruskal-Wallis rank sum tests (X^2^) were used for independent variables containing three groups to test the null hypotheses that samples were from identical populations. Conover post hoc pairwise multiple comparison tests (t) with Bonferroni adjustments followed Kruskal-Wallis tests with *p*-values < 0.05 to identify which groups differed significantly.

Following initial analysis described above, post-hoc, one-tailed Wilcoxon-Mann-Whitney tests (U) were used for selective two-level variables to identify whether observed differences between them were significant. Details of all the statistical test results are provided in Additional file 3, together with their *p*-values, z-scores and effect-sizes (r). All statistical tests were performed using R version 3.3.2 statistical software [25].

## Results

### Participant characteristics

One hundred and twelve participants between the ages of 18 and 25 completed the survey. Their mean age was 21.9 (SD = 2.28). Ninety two (82.1%) were full-time students and twenty (17.9%) were non-academic employees. One hundred were recruited from the University of Nottingham, nine from a Nottinghamshire school and three were conference attendees. For fifty eight participants (51.8%), this survey was the first time they had heard of WGS. Seventy nine participants (70.5%) wanted to learn more about WGS and the human genome. The highest completed education level attained was self-reported, translated to correspond with the eight levels of the UK Visas and Immigration Qualification Level list [26], then grouped into three completed educational levels. The first included those whose highest educational level attained was secondary school education or equivalent vocational qualifications, the second level included those whose highest attainment was a degree level qualification or equivalent and the third level included those with a further degree, equivalent or higher. The STEMM group did not differ by gender, as confirmed by a chi-squared test (X^2^(1, *N* = 112) = 2.1458,*p* = 0.143). The gender breakdown for participants’ STEMM status, previous genetics courses undertaken and completed educational levels are detailed in Table 1. In the open-ended comments, most participants’ expressed interest in the topic. None commented about any difficulties with completing questions using ellipses. It was observed that participants often responded with various ellipse sizes along the 0–10 range, evidencing their differing levels of certainty for each question and supporting face validity of the method used. Thirty six participants answered the separate questions concerning the use of ellipses. When asked how using ellipses affected their ability to express their opinion, of the 25 participants’ who responded to this specific question, 20 described the method’s effect positively, whilst five commented that it was not helpful.

**Table 1:** Independent variable levels and participant numbers

Variables	Description, numbers and %		
Gender:		Female: 67 (59.8%)	Male: 45 (40.2%)
STEMM: Gender-split:		STEMM: 59 (53%) Females 31:Males 28	Non-STEMM: 53 (47%) Females 36:Males 17
Genetics Course: Gender-split:	None: 86 (76.8%) Females 46:Males 40	School: 10 (8.9%) Females 8:Males 2	University: 16 (14.3%) Females 13:Males 3
Completed Educational Level: Gender-split:	Secondary school or equiv.	1^o^ degree:	2^o^ degree:
	46 (41.1%) Females 28:Males 18	42 (37.5%) Females 23:Males 19	24 (21.4%) Females 16:Males 8

### Statistical results

#### Attitudes towards sharing one’s WGS information with relatives

**Fig. 1:**
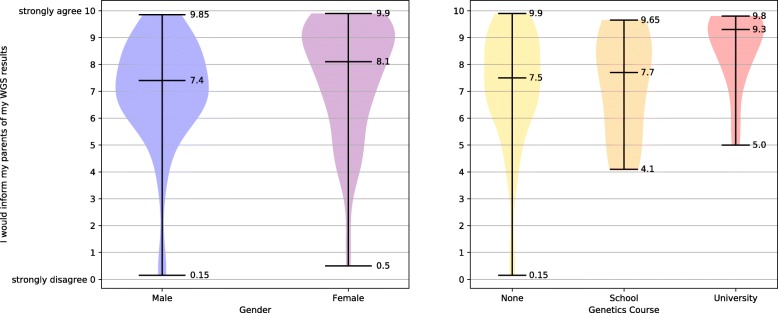
Preferences for sharing WGS results with parents

**Fig. 2:**
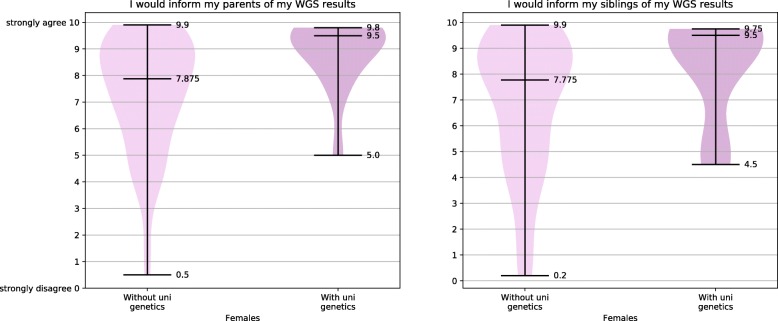
Females intention to share and University genetics education

**Fig. 3:**
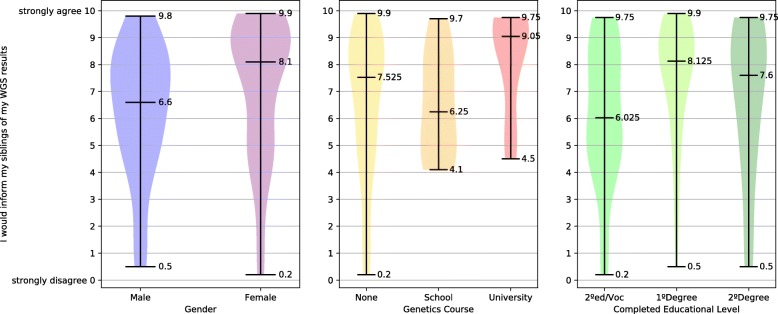
Preferences for sharing WGS results with siblings

The survey examines the relationship between participants’ gender and their intention to share their WGS results with parents (IQ7). The difference between the genders was significant, as illustrated in Fig. 1(a), with statistical results reported in Additional file 3. In relation to intention to share one’s WGS results with parents and participants’ genetics course attainment, those who had studied genetics at university had a significantly greater intention to share with parents than those who had not studied genetics at all, see Fig. 1(b). The relationship between participants’ intention to share with parents and their completed educational level was found not to be statistically significant. The relationship between intention to share results with parents and STEMM status was also found not to be statistically significant. Because intention to share with parents of participants who had previously studied genetics at university was higher than that of the female participants and due to the high ratio of females in the university-level genetics groups, females with and females without university-level genetics education were compared post-hoc for their attitudes to sharing with parents. Power for this analysis was low. Females who had studied genetics at university had a significantly greater intention to share with parents than the other females, Fig. 2(a). When the relationship between intention to share results with siblings (IQ5) and gender was examined, females were found to have significantly greater intention to share, see Fig. 3(a). When intention to share one’s WGS results with siblings was analysed in relation to participants’ prior completion of a genetics course, those who had studied genetics at university had a significantly greater intention to share their results with their siblings compared to those who had never studied genetics, as per Fig. 3(b). Examination of how the desire to share WGS results with siblings related to educational levels found that those with a secondary-school / vocational-college education had a significantly lower intention to share with siblings compared to those with a 1st degree, see Fig. 3(c). Participants’ intention to share with siblings and their STEMM status was not found to be statistically significant. Because intentions to share with siblings held by university-level genetics course participants were higher than those of female-gender group, females with and females without university-level genetics education were compared for their attitudes to sharing with siblings. Females who had studied genetics at university were found to have a significantly greater intention to share with their siblings than the other females, Fig. 2(b).

### Desire to know the WGS results of relatives

**Fig. 4:**
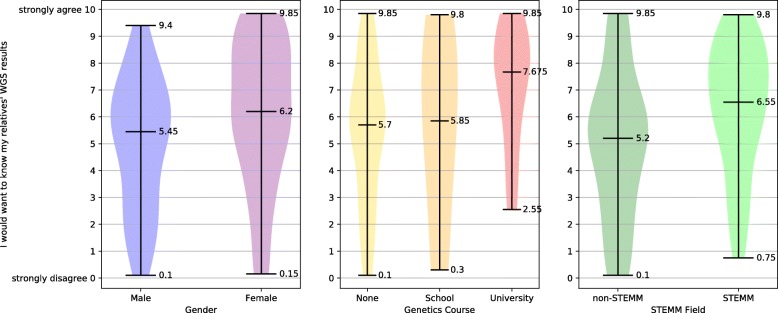
Preferences for wanting to know relatives’ WGS results

Examination of the relationship between the desire to know the WGS results of one’s relatives (IQ22) and gender found that females had a significantly greater desire to know, see Fig. 4(a). The desire to know the results of relatives and participants’ prior genetics education were compared. Those who had studied genetics at university had a significantly greater desire to know the results of relatives than those who had never studied genetics, see Fig. 4(b). The relationship between desire to know relatives’ results and completed educational level was found not to be statistically significant. The desire of those in STEMM areas to know the WGS results of relatives was significantly greater than non-STEMM, see Fig. 4(c). Because desire for relatives’ results held by University-level genetics course participants was higher than that felt by females, a comparison was made between females with and without university-level genetics education. The difference observed in their attitudes was not statistically significant.

## Discussion

This study provides new insights into the views of university-based young adults about sharing genomic results information. Other studies have explored participants’ desire to share genetic results with relatives, but most have been in the context of clinical genetics such as breast cancer [14, 16] or in paediatric settings [27, 28]. Very few genomic studies have addressed young adults’ views and even fewer considered their sharing preferences for genetic or genomic sequencing results in relation to their educational attainment or knowledge of WGS [7, 11]. Empirical studies addressing desires for genetic information resulting from a relative’s testing are rare [14, 17].

### Participant characteristics: WGS awareness, and education

The high level of WGS awareness in our participant supports the trend identified in another study that the public’s knowledge of genetics had been improving over the preceding 14 years [29]. Interest in genetic testing and previous genetics education was high among our participants compared to previous findings [29]; however, this is likely to be at least partly due to the high proportion of university students in our study.

### Attitudes towards WGS information sharing

Our study found that most young adults had a strong desire to share their WGS results with their parents and siblings, supporting previous findings [17] in which most respondents, from a wider age range, reported willingness to consent to sharing pertinent genetic information with their relatives. Participants’ responses to attitudinal questions about sharing with relatives indicated that, in terms of the TPB, many had made a positive evaluation of the potential outcome of these sharing activities when forming their attitudes. However, differences were found. Unlike another study that did not find any gender differences among young adults [7], we found that gender, genetic education, and familiarity with a STEMM field were all significant variables for the sharing preferences of young adults. Among females, those with a university-level genetics education were most willing to share their results with their relatives. Having a university-level genetics education or being female was related to higher desire to share one’s results with relatives and to want to know relatives’ results. It was found that females were significantly more willing to share their WGS results with parents and siblings. Additionally, female participants who had undertaken a university-level genetic course had significantly higher intentions to share their results with parents and siblings than females who had not studied genetics to this level (see Fig. 2). A first degree was associated with greater willingness to share WGS results with one’s siblings that was equivalent to the female gender-group (see Figs. 1 and 3). The generic measure of Completed Educational Levels was found not to be associated with a desire for relatives’ results; however, having undertaken a genetics course at university, being female or studying in a STEMM field was (see Fig. 4). These findings are contrary to another UK study [17] where lesser-educated participants had a greater desire to know their relatives results. Attitudes towards sharing WGS results with relatives are likely to be more positive for females and those who had higher levels of genetic and genomic knowledge, specifically gained through education. These groups want to share more, making them obvious conduits for genomic information within families. Information and advice designed for managing WGS results would benefit from including knowledge and attitudinal assessments that address sharing considerations. Genomic service providers may support assessment of young adults’ prior genetic education and sharing attitudes as part of personalised educational provision so the outcomes of WGS may be appreciated by the individual and others. These results raise further questions about what young adults think of sharing their results with health professionals, researchers, employers and others. Also, what genetic knowledge would be best to acquire for the purpose of undertaking elective screening using technologies such as WGS? The question of how best to support individuals to appropriately share results from genomic sequencing is also highlighted. Larger scale research is needed to further examine sharing attitudes indicated by this study. Additional research will be required to inform design and provision of educational materials that account for individuals’ pre-existing genetic knowledge and attitudes towards sharing. This study offers several new findings about young adult participants regarding attitudes and behavioural intentions towards sharing WGS results according to their gender, genetic courses undertaken, generic educational attainment level and STEMM status. These variables are likely to affect how WGS results are shared in families, affecting, for instance, relevant health promoting information which may be withheld initially, not shared or miscommunicated.

### Limitations

Limitations to this study include the sample size, its demographic make-up and elements of the methodology. As the sample size is relatively small, only the largest differences were detectable. The sample contains a large proportion of students, drawn mainly from a population of university-based individuals at the University of Nottingham. This population’s educational attainment and genetic knowledge is likely to be higher than that of the general public. The use of ellipses in collecting data is also novel in this context. This should also be considered when interpreting results. Further research is required to generalise these results.

## Conclusions

This study presents novel insights into young adults’ preferences for sharing WGS results. Gender, genetic courses, completed educational levels and STEMM-related studies featured highly as variables which could affect young adults’ behavioural intentions regarding sharing results. Those who had attained university-level genetics education responded differently from those categorised as having completed higher generic educational levels. Being female was consistently related to higher medians for sharing with parents and siblings and for desire to know relatives’ results. However those who had undertaken a university-level genetics course had even higher intentions to share with parents and with siblings, as well as having a greater desire to know relatives’ results than other categorical groups. Additionally, those females who had previously undertaken a university-level genetics course had a significantly greater intention to share their results with their parents and their siblings compared to the other females surveyed. This highlights the potential of genetics education to increase sharing intentions, even for those who already have high intentions, such as females. Further studies addressing gender, genetics education and other measures of genetic knowledge and familiarity are needed to further explore the influence each has on attitudes to sharing WGS results. In order to address their preparedness to manage and share WGS results, considerations for young adults’ should include gender, genetic knowledge, attitudes to sharing and other constructs that form behavioural intentions to receiving and sharing WGS results. Such considerations can guide how educational materials and results reports are designed and presented. Improvements to information given about sharing genomic information should encourage individuals to act both for their own and their relatives’ benefit. If results are presented to young adults in an accessible format, tailored to their developing knowledge and designed with information sharing in mind, they may be better understood, acted upon and more effectively shared. Despite the limitations identified above this study has highlighted variables that require further research to better understand how to improve attainment of knowledge and results’ sharing to benefit those involved. As WGS services and systems are designed, developed and offered to young adults, the importance of these variables will become increasingly apparent to the translation of genomic results and improved health and care.

## Additional files

Additional file 1: Whole Genome Sequencing Study: My Genomic Life Survey. This version has been refined for use with a larger participant population. It contains all items necessary to reproduce the survey. (PDF 646 kb)

Additional file 2: Dataset for Whole Genome Sequencing Study: My Genomic Life. It provides all data necessary to reproduce the results from the survey. (CSV 2 kb)

Additional file 3: Statistical Results. It contains all statistical tests undertaken for this study. (DOCX 24 kb)

## Abbreviations

1^°^: first degree (i.e. Bachelor’s level degree)
2^°^: further or second degree (i.e. Master’s level degree,)
STEMM: science technology engineering maths and medicine
TPB: theory of planned behaviour
WGS: whole-genome sequencing
