# Supplementary material for: Young adults’ attitudes to sharing whole-genome sequencing information: a university-based survey
Source: BMC Med Genomics. 2019 Apr 16;12:55. doi: 10.1186/s12920-019-0499-2 (PMC6469029; doi:10.1186/s12920-019-0499-2)
Supplement: Supplementary file 1 — Whole Genome Sequencing Study: My Genomic Life Survey. This version has been refined for use with a larger participant population. It contains all items necessary to reproduce the survey. (PDF 646 kb) [file 12920_2019_499_MOESM1_ESM.pdf]

Whole Genome Sequencing Study: My Genomic Life Survey

The University of Nottingham
UNITED KINGDOM · CHINA · MALAYSIA

Welcome to this survey designed to explore your views about receiving and sharing information from **Whole Genome Sequencing (WGS), the process of sequencing a person's entire DNA**. Analyses are performed on the sequence to provide results on various health conditions. You may fill in this survey electronically by using "text comment" or "draw free form" functions found in the "Comment" menu, (see the top right of this PDF's toolbar) and return completed survey with your consent form and prize draw coupon by e-mail to pepita.stringer@nottingham.ac.uk OR you may complete printed forms. Completed consent form, survey and prize draw coupon may be returned by post or scanned and emailed. Postal address: Pepita Stringer, Room B38, School of Computer Science, University of Nottingham, Jubilee Campus, Wollaton Road, Nottingham England NG8 1BB
If you have any questions, please get in touch with Pepita by email.

1. How did you first become aware of WGS? Tick all boxes that apply to you.

   a. Through this study ☐
   b. On the Internet ☐
   c. A video on the Internet ☐
   d. In a movie ☐
   e. On TV ☐
   f. A printed magazine or newspaper ☐
   g. A book ☐
   h. Academic journal article ☐
   i. A friend ☐
   j. A family member ☐
   k. A health professional ☐
   l. At school ☐
   m. At university ☐

   If you answered "Yes" to any of the above, please describe an example (or more).

   ____

   ____

   ____

2. Since hearing or reading about WGS, have you looked for more information about it?

   ☐ Yes ☐ No

3. If you answered “Yes” to Question 2: Where did you seek additional information?
(please tick as many options from the following that apply to you)

   a. Web links from study’s information sheet ☐
   b. Academic journals ☐
   c. Books or other printed materials ☐
   d. Other internet sources ☐
   e. Friends ☐
   f. Family members ☐
   g. Work colleagues ☐
   h. Health professionals ☐
   i. Other, please specify ____

Please answer the following questions about the human genome. It is not expected that you have all the answers.

4. Genes come in pairs, with one copy inherited from each parent.

   ☐ I don’t know ☐ false ☐ true

5. The chromosomes of men and women are similar except for one pair.

   ☐ I don’t know ☐ false ☐ true

6. For some disorders to be inherited, a mutation must come from both parents.

   ☐ I don’t know ☐ false ☐ true

7. Males and females have the same number of chromosomes.

   ☐ I don’t know ☐ false ☐ true

8. A gene is a disease.

   ☐ I don’t know ☐ false ☐ true

9. Parents with no sign of ill-health can have a child with an inherited disease.

   ☐ I don’t know ☐ false ☐ true

10. The carrier of a disease gene may be completely healthy.

☐ I don't know ☐ false ☐ true

11. Some genetic conditions express themselves later in adult life

☐ I don't know ☐ false ☐ true

12. How many pairs of chromosomes do humans have?

☐ a. 23
☐ b. 24
☐ c. 27
☐ d. 28
☐ e. I don't know

13. In DNA, the Adenine nucleotide bonds with which nucleotide to form a base pair?

☐ a. Guanine
☐ b. Cytosine
☐ c. Thymine
☐ d. Uracil
☐ e. I don't know

14. Approximately how many protein-encoding genes do humans have?

☐ a. 20,000 to 25,000
☐ b. 35,000 to 40,000
☐ c. 70,000-75,000
☐ d. More than 100,000
☐ e. I don't know

For the following questions, please draw an ellipse on the scale, of any size you choose. EXAMPLE 1

I know a good route for cycling from my office to my home.

NOT AT ALL CONFIDENT

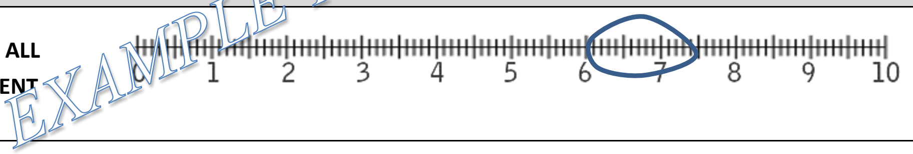

DEFINITELY CONFIDENT

EXAMPLE 2: To correct a mistake, make crosses at both sides of an incorrect ellipse (6 - 8), then draw a new one.

I know a good route for walking from my office to my home.

NOT AT ALL CONFIDENT

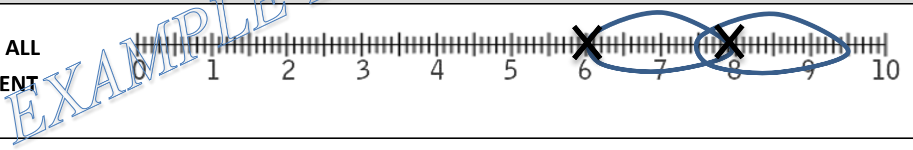

DEFINITELY CONFIDENT

Draw your ellipses as you see fit for each of the statement below.

1. I am confident I can find information on whole genome sequencing (WGS).

STRONGLY DISAGREE	0 1 2 3 4 5 6 7 8 9 10	STRONGLY AGREE

2. I am confident I would understand the relevant information about results from a WGS analysis.

STRONGLY DISAGREE	0 1 2 3 4 5 6 7 8 9 10	STRONGLY AGREE

3. I think the development of WGS is a medical progress which may have positive impact

STRONGLY DISAGREE	0 1 2 3 4 5 6 7 8 9 10	STRONGLY AGREE

4. I approve of using WGS for better management of diseases.

STRONGLY DISAGREE	0 1 2 3 4 5 6 7 8 9 10	STRONGLY AGREE

5. I would inform my siblings about the results of my WGS analysis.

STRONGLY DISAGREE	0 1 2 3 4 5 6 7 8 9 10	STRONGLY AGREE

6. I would inform my children about the results of my WGS analysis.

STRONGLY DISAGREE	0 1 2 3 4 5 6 7 8 9 10	STRONGLY AGREE

7. I would inform my parents about the results of my WGS analysis.

STRONGLY DISAGREE	0 1 2 3 4 5 6 7 8 9 10	STRONGLY AGREE

8. I would want to know if I had a hereditary disease.

STRONGLY DISAGREE	0 1 2 3 4 5 6 7 8 9 10	STRONGLY AGREE

9. I think having the results of my WGS analysis would help me take more responsibility for my health.

STRONGLY DISAGREE 0 1 2 3 4 5 6 7 8 9 10 STRONGLY AGREE

10. Knowing the results from WGS analysis could change a person's future.

STRONGLY DISAGREE 0 1 2 3 4 5 6 7 8 9 10 STRONGLY AGREE

---

11. I am concerned about possible consequences the WGS results may have on insurance policies for health, travel or life.

STRONGLY DISAGREE 0 1 2 3 4 5 6 7 8 9 10 STRONGLY AGREE

12. I only want to know about diseases that can be treated.

STRONGLY DISAGREE 0 1 2 3 4 5 6 7 8 9 10 STRONGLY AGREE

13. I would prefer <u>not to undertake</u> WGS.

STRONGLY DISAGREE 0 1 2 3 4 5 6 7 8 9 10 STRONGLY AGREE

14. I want my WGS to help me learn about my ancestry and my family tree.

STRONGLY DISAGREE 0 1 2 3 4 5 6 7 8 9 10 STRONGLY AGREE

15. The idea of WGS frightens me.

STRONGLY DISAGREE 0 1 2 3 4 5 6 7 8 9 10 STRONGLY AGREE

---

16. I would like to have my own WGS done.

STRONGLY DISAGREE 0 1 2 3 4 5 6 7 8 9 10 STRONGLY AGREE

17. I would want to receive a report that explains the results from my WGS.

STRONGLY DISAGREE	0 1 2 3 4 5 6 7 8 9 10	STRONGLY AGREE

18. I would want to receive the raw data from my WGS.

STRONGLY DISAGREE	0 1 2 3 4 5 6 7 8 9 10	STRONGLY AGREE

19. I would consult a doctor, nurse, or counsellor before undertaking a WGS.

EXTREMELY UNLIKELY	0 1 2 3 4 5 6 7 8 9 10	EXTREMELY LIKELY

20. I like the idea of purchasing WGS services over the Internet.

STRONGLY DISAGREE	0 1 2 3 4 5 6 7 8 9 10	STRONGLY AGREE

21. My relatives would want to know about my WGS results.

STRONGLY DISAGREE	0 1 2 3 4 5 6 7 8 9 10	STRONGLY AGREE

22. I would want to know the WGS results of my relatives.

STRONGLY DISAGREE	0 1 2 3 4 5 6 7 8 9 10	STRONGLY AGREE

23. I would consider knowing my WGS analysis as:

HARMFUL	0 1 2 3 4 5 6 7 8 9 10	BENEFICIAL

24. I would consider knowing my WGS analysis as:

WORTHLESS	0 1 2 3 4 5 6 7 8 9 10	VALUABLE

25. I consider myself…
VERY UNHEALTHY 0 1 2 3 4 5 6 7 8 9 10 VERY HEALTHY

Please fill in the blanks and make a tick in the circles ✓ that apply to you:

26. Please state your age: I am___________years old

27. Please state your gender: I am ____________________

28. Have you studied genetics as part of a biology course?

○ no ○ yes (at school) ○ yes (at university)

29. Have you been on a course specifically about genetics?

○ no ○ yes (at school) ○ yes (at university)

30. What is your highest completed level of education? ____________________________

31. Are you employed?
○ no ○ yes, part-time ○ yes, full-time
If you are employed, what is your current role(s)?_________________________________

32. Are you a student?
○ no ○ yes, part-time ○ yes, full-time
If you are a student, what is your field of study?___________________________________

33. Do you teach or do research?
○ no ○ yes

If you teach or do research, what is your field?

____

34. What country have you mainly resided in for the last 6 months?

____

35. Would you want your genomic data to be available to wider health research studies?

○ no ○ yes, but only if I'm not re-identifiable ○ yes, if I can be re-identified and contacted
○ other response, please describe____

36. Would you like to learn more about WGS or the human genome?
○ no ○ yes

Please make comments____

____

____

____

This survey used the following method to gather your opinions, see the example below:

Please draw an ellipse on the scale to reflect your views.

I like using this method to answer questions about my opinion.		
STRONGLY DISAGREE	0 1 2 3 4 5 6 7 8 9 10	STRONGLY AGREE

36. Please describe how the method used in this survey affected your ability to express your opinions.

____

____

Many surveys use the following format for gathering opinions:

Please click the dot to select your response from those below:

I like using this method to answer questions about my opinion.

○ Strongly Disagree ○ Disagree ○ Slightly Disagree ○ Neither ○ Slightly Agree ○ Agree ○ Strongly Agree

37. Would you have been able to express yourself better using this method?

____

____

38. Please select words below that reflect your opinion of the method used in this survey (select all that apply).

○ Realistic	○ Confusing	○ Intuitive
○ Easy	○ Time-efficient	○ Correct
○ Natural	○ Vague	○ Cryptic
○ Clear	○ Difficult	○ Time-consuming

Please write three words below to describe your thoughts about using the method above to answer the questions in this survey.

- ___________________________
- ___________________________
- ___________________________

Completed **consent form and survey** to be returned by email to pepita.stringer@nottingham.ac.uk, by post to Pepita Stringer, School of Computer Science, University of Nottingham, Wollaton Road, Nottingham England NG8 1BB, in person or as directed. **Thank you for your participation, it is very much appreciated!**
