# Supplementary material for: Young adults’ attitudes to sharing whole-genome sequencing information: a university-based survey
Source: BMC Med Genomics. 2019 Apr 16;12:55. doi: 10.1186/s12920-019-0499-2 (PMC6469029; doi:10.1186/s12920-019-0499-2)
Supplement: Supplementary file 3 — Statistical Results. It contains all statistical tests undertaken for this study. (DOCX 24 kb) [file 12920_2019_499_MOESM3_ESM.docx]

| **n = 112** | **Statistical Test Results** | | | | |
| --- | --- | --- | --- | --- | --- |
| **Statistical Test ⇒** | Mann Whitney (U) | Mann Whitney (U) | Kruskal-Wallis (X^2^) | Kruskal-Wallis (X^2^) | Mann Whitney (U) – post-hoc |
| **Single test alpha Mult-test alpha** | α = 0.05  α = 0.001 | α = 0.05  α = 0.001 | α = 0.05  α = 0.001 | α = 0.05  α = 0.001 | α = 0.05  α = 0.0166 |
| **Variables** | **Gender (f, m)** | **STEM (n, y)** | **Completed Ed-Level (grouped)** | **Genetics course (none, school, university)** | **Post-hoc test: Females without and females with university-level genetics course** |
| **sharing WGS results with parents (Q7)** | **U = 1856**  **p = .0388**  *****  **z = -2.068**  **r = -0.19541** | U = 1288  p = 0.108  z = 1.605  r = 0.151658 | X^2^(2) = 3.5372  p = 0.1706  ns | **X^2^(2) = 8.273**  **p = 0.01598**  *****  **-------------------**  **Conover post-hoc test result for University x none**  **t(110) = 2.906**  **p < 0.05**  **r = 0.26701** | **U = 179.5**  **p = .00336**  ******  **z = -2.71121**  **r = 0.331227** |
| **sharing WGS results with siblings (Q5)** | **U = 1849.5**  **p = 0.04258**  *****  **z = -2.03**  **r = -0.19182** | U = 1287  p = 0.107  z = 1.611  r = 0.152225 | **X^2^(2) = 6.1039**  **p = 0.04727**  *****  **-------------------**  **Conover post-hoc test result for Secondary School x 1^st^ degree**  **t(110) = 2.518, p < 0.05**  **r = 0.23344** | **X^2^(2) = 6.8617**  **p = 0.03236**  *****  **-------------------**  **Conover post-hoc test result for University x none**  **t(110) = 2.529,**  **p < 0.05**  **r = 0.23441** | **U = 204.5**  **p = .01044**  *****  **z = -2.31484**  **r = 0.282803** |
| **wanting to know WGS results of a relative (Q22)** | **U = 1912**  **p = 0.0164**  *****  **z = -2.404**  **r = -0.22716** | **U = 1104.5**  **p = 0.00753**  ******  **z = 2.675**  **r = 0.252764** | X^2^(2) = 1.723  p = 0.4225  ns | **X^2^(2) = 6.2077**  **p = 0.04488**  *****  **------------------**  **Conover post-hoc test result for University x none**  **t(110) = 2.533,**  **p < 0.05**  **r = 0.23476** | U = 254  p = .06301  ns  z = -1.53001  r = 0.186920 |

| **Interpretation of effect size (r)** | |
| --- | --- |
| **r = 0.1** | **Small effect** |
| **r = 0.3** | **Medium effect** |
| **r = 0.5** | **Large effect** |
